# The modified capsular arthroplasty for young patients with developmental dislocation of the hip

**DOI:** 10.1093/jhps/hnad017

**Published:** 2023-06-13

**Authors:** Zhendong Zhang, Dianzhong Luo, Hui Cheng, Hong Zhang, Jianli Zhang, Ningtao Ren, Yong Li, Reinhold Ganz

**Affiliations:** Senior Department of Orthopedics, The Fourth Medical Center of Chinese PLA General Hospital, 51 Fucheng Road, Beijing 100048, China; Senior Department of Orthopedics, The Fourth Medical Center of Chinese PLA General Hospital, 51 Fucheng Road, Beijing 100048, China; Senior Department of Orthopedics, The Fourth Medical Center of Chinese PLA General Hospital, 51 Fucheng Road, Beijing 100048, China; Senior Department of Orthopedics, The Fourth Medical Center of Chinese PLA General Hospital, 51 Fucheng Road, Beijing 100048, China; Senior Department of Orthopedics, The Fourth Medical Center of Chinese PLA General Hospital, 51 Fucheng Road, Beijing 100048, China; Senior Department of Orthopedics, The Fourth Medical Center of Chinese PLA General Hospital, 51 Fucheng Road, Beijing 100048, China; Senior Department of Orthopedics, The Fourth Medical Center of Chinese PLA General Hospital, 51 Fucheng Road, Beijing 100048, China; Faculty of Medicine, University of Bern, Hochschulstrasse, Bern 3012, Switzerland

## Abstract

The present study aimed to investigate the clinical results of the modified Codivilla–Hey Groves–Colonna capsular arthroplasty in the treatment of young patients with developmental dislocation of the hip. We retrospectively evaluated 90 patients (92 hips) who underwent the modified capsular arthroplasty from June 2012 to June 2021. Hips were evaluated using the modified hip Harris score (mHHS), the Western Ontario and McMaster Universities Osteoarthritis Index (WOMAC) score and the 12-item International Hip Outcome Tool (iHOT-12). The Tönnis osteoarthritis grade and the Severin classification system were used to assess the radiographic outcomes. The average age was 15.7 years (range: 8–26 years). The mean pre-operative mHHS, the WOMAC score and the iHOT-12 score were 83.03, 14.05 and 52.79, respectively. The patients were followed for a mean of 41.1 months (range: 12.1–120.9 months). The patients had a mean mHHS of 83.61 (range: 31.2–97), a WOMAC score of 16.41 (range: 0–51) and an iHOT-12 score of 64.81 (range: 12.9–98.2) at the final follow-up. Capsular thickness had a positive predication on the final functional outcomes. The excellent/good rate of radiological reduction was 79.3%. More than 60% of patients had no/slight osteoarthritis. A total of 54 hips (58.7%) had superior radiographic outcomes. The risk factors for inferior radiographic outcomes were capsular quality (odds ratio [OR]: 0.358, 95% confidence interval [CI]: 0.113–0.931) and capsular thickness (OR: 0.265, 95% CI: 0.134–0.525). Joint stiffness was the most common complication (14.1%). We confirmed the efficacy of this procedure in the treatment of developmental hip dislocation. Patients with poor capsular quality are not suitable for this procedure. With suitable selection according to indications, this procedure can restore the hip rotation center with a low incidence of femoral head necrosis or severe osteoarthritis.

## INTRODUCTION

Developmental dislocation of the hip (DDLH) is one of the most serious deformities of the hip. It is characterized by complete dislocation of the femoral head from the acetabulum. Total hip arthroplasty (THA) is the ultimate treatment for osteoarthritis caused by DDLH [1]. However, for active young people and adolescents, the revision-free survival rate is lower than older patients [2, 3]. Therefore, hip preservation surgery is of great importance for adolescents and older children. Due to the balanced bilateral muscle strength and symmetrical bone structure, secondary degenerative changes in the hip joint in patients with bilateral DDLH are usually absent at an early age. Generally, no surgical treatment is needed when patients are young or there is no sign of osteoarthritis. However, for young patients with unilateral DDLH, the lower limb length discrepancy is obvious, which not only affects the appearance but also, more importantly, causes secondary scoliosis, low back pain, knee valgus deformity or other serious intractable problems [4–6]. Therefore, early treatment is essential.

Pediatric orthopedic surgeons can address unilateral DDLH by open reduction or reshaped acetabulum by pelvic osteotomy or shelf augmentation under the age of eight. However, reduction of the femoral head can be difficult for older children and adolescents due to the incapability of the abnormal acetabulum to be reshaped to match the femoral head. Thus, unilateral DDLH cannot be properly treated with various joint-preserving procedures.

Capsular arthroplasty is a hip preservation surgery used to treat developmental hip dislocation by wrapping the capsule around the femoral head and relocating it into the newly reamed acetabulum at its anatomical level. This concept was first proposed by Codivilla in 1901 [7], and in 1926, Hey Groves described a similar procedure [8]. It was named capsular arthroplasty by Colonna in a series of reports [9, 10]. Early capsular arthroplasty was described as a two-stage procedure. In the first stage, the soft tissue was released, followed by continuous traction of the lower limb to reduce the difficulty of subsequent reduction. Then, in the second stage, capsular arthroplasty was performed. Due to the technical difficulty and the high incidence of complications of two-stage capsular arthroplasty, including femoral head necrosis and joint stiffness, this surgical procedure has been gradually abandoned.

After research on the anatomy of the blood supply of the femoral head, Ganz *et al.* [11] in 2012 reported a modified Codivilla–Hey Groves–Colonna capsular arthroplasty, hereinafter referred to as the modified capsular arthroplasty, a one-stage procedure involving surgical hip dislocation and capsular arthroplasty. Ganz *et al.* [11] reported the clinical results of nine patients. The average Harris hip score of seven patients was 84 at the final follow-up. No femoral head osteonecrosis occurred. Therefore, with the successful modified capsular arthroplasty, the hip can be preserved, or THA can be postponed. In addition, this procedure reconstructs the acetabulum and reduces the femoral head to the true acetabulum, which simplifies the difficulty of THA and reduces the risk of complications, such as nerve traction [12–14].

To date, there are few reports in the literature regarding the modified capsular arthroplasty for the treatment of DDLH. Since June 2012, nearly 100 patients with DDLH have been treated in The Fourth Medical Center of Chinese PLA General Hospital. Never before has the literature reported this kind of case on such a scale. Our purposes were to investigate the clinical results, analyze the prognostic factors and summarize the complications of the modified capsular arthroplasty.

## MATERIALS AND METHODS

The modified capsular arthroplasty procedure was initially performed in our department under the guidance of Ganz. Therefore, all surgeries were conducted by the standard method described previously by Ganz [11]. After approval by the institutional review board of The Fourth Medical Center of Chinese PLA General Hospital, we retrospectively evaluated all patients who underwent the modified capsular arthroplasty from June 2012 to June 2021. The general characteristics, surgical details and follow-up data of patients with a minimum 1-year follow-up were reviewed. Complications occurring at any time throughout the follow-up period were recorded. Four patients (4 hips) were excluded due to the loss of follow-up, leaving 90 patients (92 hips) enrolled.

Patients’ demographic and surgical data are shown in Table I. All patients were classified as type II or III according to the Hartofilakidis classification system [6]. Only two patients received bilateral procedures, which were both performed at an early stage (2012–13).

**Table I.** Patients’ demographic and surgical data

*Variable*	*Values*
No. of patients	90 patients (92 hips)
Sex	
Female	73 patients (74 hips)
Male	17 patients (18 hips)
Age (years)	15.7 ± 4.8 (range: 8–26)
Body mass index (kg/m^2^)	19.1 ± 3.1 (range: 12.6–27.4)
Side	
Left	50 hips (54.3%)
Right	42 hips (45.7%)
Comorbidities (*n* = 6)	
Sequelae of cerebral palsy	3 cases
Meningomyelocele	1 case
Spina bifida	1 case
Down’s syndrome	1 case
Previous surgery (*n* = 26)	
Open reduction and/or osteotomy	12 cases
Spica cast or Pavlik harness	14 cases
Hartofilakidis	
Type II	32 patients (34 hips)
Type III	58 patients (58 hips)
Concurrent procedures	
Proximal femoral osteotomy	80 patients (81 hips)
Femoral head reduction osteotomy	2 patients (2 hips)
Follow-up time (m)	41.1 ± 23.5 (range: 12.1–120.9)

Pre-operatively, all patients completed the modified Harris hip score (mHHS) questionnaire and Western Ontario and McMaster Universities Osteoarthritis Index (WOMAC) score, both of which are reliable tools for assessing hip pain and functional outcomes. However, for older children or young and adolescent patients with DDLH, a limp is usually presented as the chief complaint rather than pain. Therefore, patients with unilateral developmental hip dislocation may have both a severe limp and a high mHHS. The 12-item International Hip Outcome Tool (iHOT-12) was used to supplement the mHHS and WOMAC because it has an excellent performance in evaluating the hip function of patients treated with hip-preserving surgery [15]. Follow-up was conducted first at 6 weeks post-operatively, then regularly every 3 months for 1 year and every 6 months thereafter. For patients who were unable to visit, the questionnaires were sent by mobile phone. Thus, we obtained consecutive functional scores for each patient.

At each follow-up visit, both the Tönnis osteoarthritis grade [16] and the Severin classification system [17] were used to assess the radiographic outcomes, which were confirmed by at least two senior orthopedic surgeons twice with an interval of 4 weeks between scorings. Tönnis Grade ≥2 and/or Severin Class ≥III were considered inferior radiographic outcomes.

The capsule tissue, which is interposed between the femoral head and the newly reamed acetabulum, is also attached to the weight-bearing area of the new hip joint and may be transformed into fibrocartilage post-operatively. The capsular quality was regarded as good when the nuclear magnetic resonance signal was continuous and uniform (Fig. 1). The outermost superior–lateral capsular thickness was measured on magnetic resonance imaging pre-operatively, as this part generally lies in the weight-bearing area of the new hip joint. In addition, we also analyzed the general characteristics, pre- and post-operative femoral anteversion, mean femoral head diameter, new socket diameter and follow-up time to identify the risk factors for inferior outcomes.

**Fig. 1.**
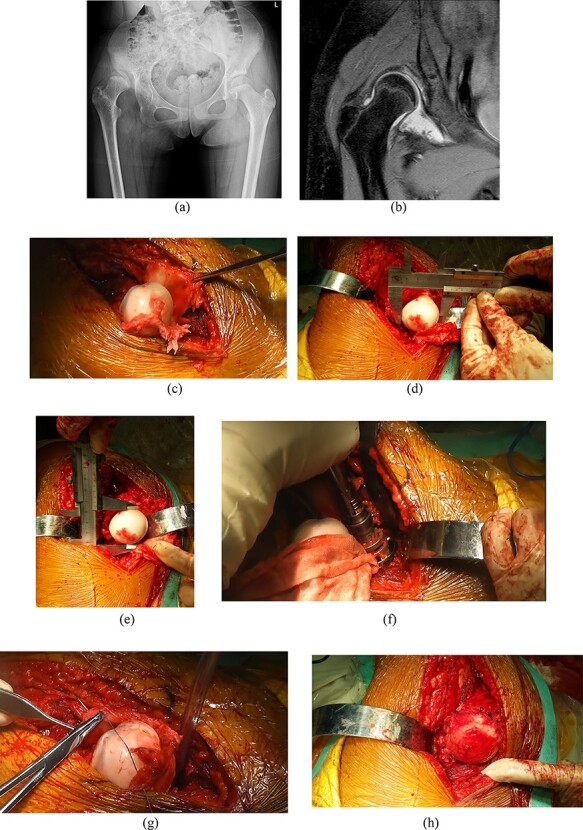
The modified capsular arthroplasty in a 13-year-old female patient with DDLH (iHot 62.3, WOMAC 9 and mHHS 74). (**A**) Pre-operative anterior-posterior radiograph shows the Hartofilakidis Type III dislocation of the hip. (**B**) Pre-operative MRI shows the good quality of capsular. The outermost superior–lateral capsular thickness is 5.3 mm. (**C**–**H**) Intraoperative pictures show the brief steps of the surgery. (C) The femoral head is dislocated and all capsular connections with the acetabular rim are sectioned. (D) and (E) show the measurements of anteroposterior and medial–lateral femoral head diameters. (F) shows the reaming of the acetabular socket. (G) and (H) show the suture of the capsule over the head. X-ray immediately after surgery (**I**), 2 years post-operatively (**J**) and 6 years post-operatively (**K**) (iHot 91.1, WOMAC 5, mHHS 94).

**Fig. 1.**
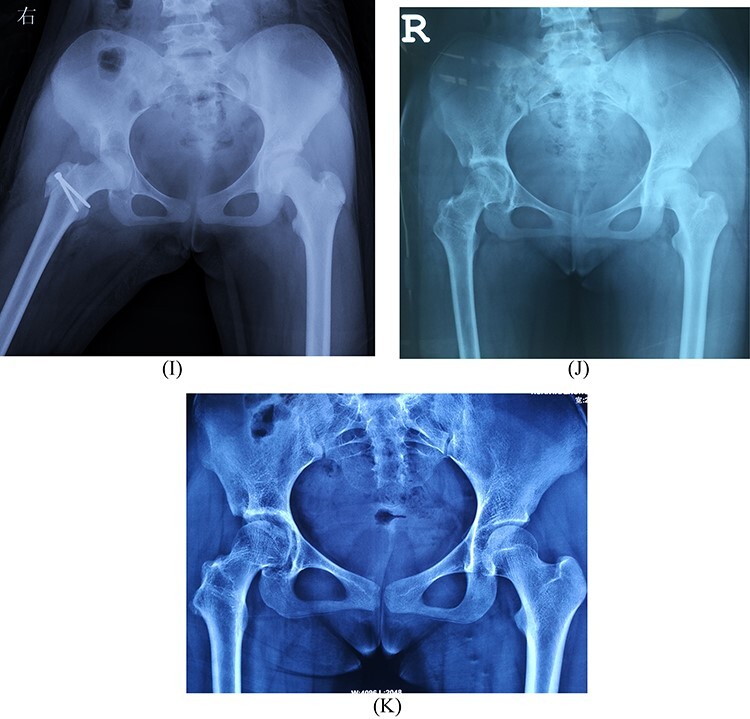
(Continued)

### Statistical analysis

Categorical variables are reported as percentages, and continuous variables are reported as the mean and standard deviation (SD) if the data followed a normal distribution. The pre- and post-operative functional scores were compared by paired samples *t-*tests. To determine the risk factors for inferior radiographic outcomes, we performed a logistic regression analysis. First, we used *P* < 0.15 in univariate analysis to identify potential significant variables and then analyzed these potential variables by multivariate analysis. The odds ratio (OR) with 95% confidence interval (CI) was obtained for each variable. Multiple linear regression analysis was also performed to identify predictive factors for functional outcomes. SPSS Statistics 29.0.0 (IBM) was used to perform the statistical analysis. *P* < 0.05 was considered to be significant.

## RESULTS

### Functional outcomes

Analysis of the differences between the pre-operative hip functional scores and those at the final follow-up showed a significant improvement in the iHOT-12. No significant difference was found either in the mHHS or in the WOMAC (Table II). The mHHS was ≥90 for 29 hips (31.5%) and between 70 and 90 for 55 hips (59.8%), but it was <70 in 8 hips (8.7%).

**Table II.** Functional outcomes

	*Pre-operative*	*Final follow-up*	*P-value*
iHOT-12	52.79 ± 17.93	64.81 ± 19.09	**<0.001**
mHHS	83.03 ± 11.84	83.61 ± 11.40	0.686
WOMAC	14.05 ± 13.69	16.41 ± 14.82	0.134

The bold values indicate statistically significant results.

The multiple linear regression analysis showed that all regression equations between functional outcomes and corresponding predictive factors were significant (WOMAC score: *F *= 3.581, *P* < 0.001; mHHS: *F *= 3.045, *P* < 0.001; iHOT-12 score: *F *= 4.028, *P* < 0.001). The capsular thickness (*β *= −0.446, *P* < 0.001), mean femoral head diameter (*β *= −0.228, *P* = 0.041) and new socket diameter (*β *= −0.291, *P* = 0.029) had a negative predictive value for the final WOMAC score (Table III). The capsular thickness (*β *= 0.379, *P* = 0.002) and follow-up time (*β *= 0.242, *P* = 0.016) positively predicted the final mHHS (Table IV). The capsular thickness (*β *= 0.583, *P* < 0.001), ean femoral head diameter (*β *= 0.343, *P* = 0.008) and new socket diameter (*β *= 0.343, *P* = 0.009) positively predicted the final iHOT-12 score (Table V).

**Table III.** Predictive factors for the final WOMAC score

*Variables*	*B*	*β*	*t*	*P*	*F*	*Adapted R^2^*
Female	−1.180	−0.032	−0.276	0.783	3.581	0.451
Age	−0.377	−0.122	−0.977	0.332		
Height	1.277	0.898	1.869	0.066		
Weight	−1.793	−1.176	−1.324	0.190		
BMI	4.113	0.848	1.310	0.194		
Left side	3.671	0.124	1.271	0.208		
Comorbidity	4.379	0.084	0.801	0.426		
Treatment history	1.005	0.031	0.315	0.754		
Hartofilakidis Type III	3.225	0.106	1.032	0.305		
Pre-operative femoral anteversion	−0.205	−0.182	−1.414	0.161		
Combined with femoral osteotomy	2.251	0.050	0.391	0.697		
Post-operative femoral anteversion	0.474	0.184	1.736	0.087		
Capsular quality	−2.737	−0.093	−0.869	0.388		
Capsular thickness	−6.485	−0.446	−3.968	**<0.001**		
Mean femoral head diameter	−0.645	−0.228	−1.772	**0.041**		
New socket diameter	−0.748	−0.291	−2.231	**0.029**		
Follow-up time	0.016	0.024	0.253	0.801		

The bold-faced entries indicate statistically significant results.

**Table IV.** Predictive factors for the final mHHS

*Variables*	*B*	*β*	*t*	*P*	*F*	*Adapted R^2^*
Female	2.088	0.073	0.614	0.541	3.045	0.412
Age	−0.223	−0.093	−0.725	0.471		
Height	−0.272	−0.249	−0.500	0.618		
Weight	0.403	0.343	0.373	0.710		
BMI	−1.031	−0.276	−0.412	0.681		
Left side	−1.119	−0.049	−0.486	0.628		
Comorbidity	−4.745	−0.118	−1.090	0.279		
Treatment history	−1.595	−0.064	−0.627	0.533		
Hartofilakidis Type III	−0.239	−0.010	−0.096	0.924		
Pre-operative femoral anteversion	−0.191	−0.220	−1.654	0.102		
Combined with femoral osteotomy	7.340	0.210	1.601	0.114		
Post-operative femoral anteversion	0.064	0.032	0.294	0.769		
Capsular quality	1.347	0.059	0.537	0.593		
Capsular thickness	4.246	0.379	3.260	**0.002**		
Mean femoral head diameter	−0.430	−0.198	−1.481	0.143		
New socket diameter	0.387	0.195	1.447	0.152		
Follow-up time	0.125	0.242	2.469	**0.016**		

The bold-faced entries indicate statistically significant results.

**Table V.** Predictive factors for the final iHOT-12 score

*Variables*	*B*	*β*	*t*	*P*	*F*	*Adapted R^2^*
Female	5.621	0.117	1.051	0.297	4.028	0.481
Age	−0.460	−0.115	−0.951	0.345		
Height	0.625	0.341	0.730	0.468		
Weight	−1.767	−0.900	−1.041	0.301		
BMI	3.786	0.606	0.962	0.339		
Left side	0.189	0.005	0.052	0.958		
Comorbidity	4.834	0.072	0.706	0.482		
Treatment history	−1.309	−0.031	−0.327	0.745		
Hartofilakidis Type III	−0.617	−0.016	−0.158	0.875		
Pre-operative femoral anteversion	−0.032	−0.022	−0.177	0.860		
Combined with femoral osteotomy	1.383	0.024	0.192	0.848		
Post-operative femoral anteversion	0.103	0.031	0.300	0.765		
Capsular quality	−1.681	−0.044	−0.426	0.671		
Capsular thickness	10.925	0.583	5.333	**<0.001**		
Mean femoral head diameter	1.248	0.343	2.735	**0.008**		
New socket diameter	1.136	0.343	2.703	**0.009**		
Follow-up time	0.104	0.120	1.305	0.196		

The bold-faced entries indicate statistically significant results.

### Radiographic outcomes

Thirty-seven hips (40.2%) were identified as Tönnis Grade 0, 22 hips (23.9%) as Grade 1, 21 hips (22.8%) as Grade 2 and 12 hips (13.0%) as Grade 3. Fifty hips (54.3%) were classified as Severin Class I, 23 hips (25.0%) as Class II, 16 hips (17.4%) as Class III and the remaining 3 hips (3.3%) as Class IV. Four cases (4.3%) of femoral head necrosis occurred. A total of fifty-four hips (58.7%) had superior radiographic outcomes. Multivariate analysis of risk factors for inferior radiographic outcomes identified capsular quality (OR: 0.358, 95% CI: 0.113–0.931) and capsular thickness (OR: 0.265, 95% CI: 0.134–0.525) (Table VI).

**Table VI.** Univariate and multivariate analyses of risk factors for inferior radiographic outcomes (Tonnis Grade ≥2 and Severin Class ≥III) after capsular arthroplasty

	*Univariate analysis*		*Multivariate analysis*	
*Variables*	*OR (95% CI)*	*P-value*	*OR (95% CI)*	*P-value*
Female	0.883 (0.308–2.533)	0.817		
Age	1.070 (0.979–1.169)	**0.136**	0.960 (0.827–1.114)	0.592
Height	0.989 (0.950–1.029)	0.571		
Weight	1.038 (0.992–1.087)	**0.106**	0.969 (0.860–1.091)	0.601
BMI	1.239 (1.059–1.448)	**0.007**	1.231 (0.853–1.776)	0.267
Left side	0.889 (0.387–2.044)	0.782		
Comorbidity	4.875 (0.927–25.634)	**0.061**	1.617 (0.167–15.638)	0.678
Treatment history	0.491 (0.188–1.282)	**0.146**	0.346 (0.085–1.398)	0.136
Hartofilakidis Type III	1.490 (0.622–3.567)	0.371		
Pre-operative femoral anteversion	0.985 (0.954–1.017)	0.357		
Combined with femoral osteotomy	0.544 (0.153–1.933)	0.347		
Post-operative femoral anteversion	1.031 (0.958–1.109)	0.420		
Capsular quality	0.227 (0.092–0.562)	**0.001**	0.358 (0.113–0.931)	**0.038**
Capsular thickness	0.232 (0.128–0.420)	**<0.001**	0.265 (0.134–0.525)	**<0.001**
Mean femoral head diameter	0.945 (0.871–1.025)	0.175		
New socket diameter	1.019 (0.948–1.096)	0.608		
Follow-up time	1.008 (0.989–1.027)	0.429		

The bold-faced entries indicate statistically significant results.

### Reoperation and complications

One patient underwent THA after 41.5 months. Two patients underwent periacetabular osteotomy due to severe hip subluxation. Joint stiffness was the most common complication (13 cases, 14.1%). The complications are shown in Table VII.

**Table VII.** Complications

*Complications*	*Cases*	*%*
Joint stiffness	13	14.1
Femoral head necrosis	4	4.3
Subluxation	4	4.3
Delay union of greater trochanter	1	1.1
Femoral neck fracture after removal of internal fixation	1	1.1
Femoral shaft fracture after removal of internal fixation	1	1.1
Infection	1	1.1

### Gait

**Fig. 2.**
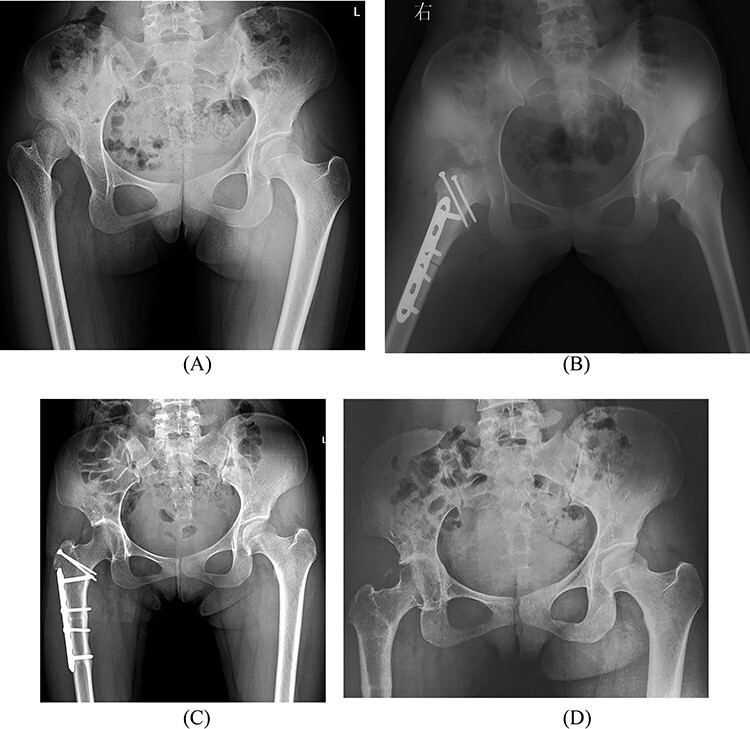
The modified capsular arthroplasty combined with proximal femoral derotation and shortening osteotomy in a 20-year-old female with a right hip dislocation (Hartofilakidis Type III). (**A**) Pre-operative radiograph; (**B**) post-operative radiograph; (**C**) 3 years post-operatively and (**D**) 6 years and 5 months post-operatively. The final radiograph shows the joint space is normal.

Sixty-eight patients (69 hips) underwent both pre- and post-operative video recordings of gait. The gait analysis showed that four hips presented as absolutely normal gait (2/4 could run normally) (Fig. 2), 17 hips as a slight limp (3/17 as nearly normal), 21 hips as a moderate limp and 27 hips as a severe limp. The limp was improved in 40 hips, and only 2 hips showed deteriorative gait. The remaining 27 hips showed consistent gait compared with the pre-operative gait. One patient with cerebral palsy who was incapable of walking pre-operatively could walk with the help of a walking aid at the final follow-up. Another patient with cerebral palsy who relied on crutches pre-operatively could walk independently at the final follow-up.

## DISCUSSION

Various outcomes have been reported for early capsular arthroplasty. Chung *et al.* reported a long-term follow-up of 56 patients who underwent capsular arthroplasty by Colonna and found that excellent or good results occurred in 31 patients [12]. Pozo *et al.* reported that three-quarters of patients had an HHS of more than 80 at the final follow-up [18]. However, early studies also reported a high failure rate of this procedure, which was mainly affected by complications, including joint stiffness, femoral head necrosis, insufficient coverage and redislocation [12, 18–22]. Due to the high incidence of complications, the complexity of surgical techniques, the improvement of hip screening mechanisms and the revolution of joint replacement techniques and materials, this procedure has been gradually abandoned. However, hip screening is not conducted in most areas, especially in some remote areas. Furthermore, some of these patients do not go to the hospital until they have obvious claudication or pain symptoms. Hip preservation surgery, such as traction reduction combined with shelf augmentation or Chiari osteotomy, cannot achieve ideal reduction. The modified capsular arthroplasty, however, can restore the hip rotation center, delay or avoid THA and simplify the difficulty of subsequent THA. Using modified surgical techniques, the rate of complications can be substantially reduced. Therefore, this procedure that was once abandoned has been gradually revived.

Our results demonstrate that most patients could obtain good functional and radiographic outcomes. Seventy-four patients (80%) had an mHHS greater than 80. There was a positive correlation between prolonged follow-up time and mHHS; that is, the longer the follow-up time was, the better the joint function that could be achieved. The iHOT-12 score was also significantly improved. The excellent and good rate of radiological reduction was 79.3% (Severin Classes I and II). More than 60% of patients had no or slight osteoarthritis. The overall complication rate was significantly lower than that reported in previous literature.

According to a study by Litt and Coutelier [23], fibrocartilaginous metaplasia occurred in the interposed capsule at the weight-bearing area, which was the key to new joint formation. Histological metaplasia can be greatly influenced by the post-operative activity level and hip range of motion. The adhesion between the capsule and new socket generally occurred 2 weeks post-operation. Therefore, we recommend holding the leg by a plaster spica cast with slight abduction for 10–14 days. In addition, abducent plaster can maintain the stability of the hip, thereby reducing the incidence of hip subluxation. Post-operative passive hip motion should be constrained within certain ranges: hip flexion: <40° within 3 weeks, <60° within 6 weeks and <90° within 3 months; internal/external rotation: none within 6 weeks and <20° within 3 months; adduction: none and abduction: 15°–30° within 3 months. The range of motion continues to increase after 3 months, and the muscle force recovers gradually after strength training. Weight-bearing begins 8–10 weeks after surgery, with a load less than one-fourth of the body weight. Full weight-bearing is generally allowed at 3–6 months after surgery. Over-exercise or early weight-bearing can lead to wear and tear of new joints. Conversely, joint stiffness may occur. In our study, irregular return visits and inappropriate rehabilitation exercises may have been the causes of joint stiffness.

Osteonecrosis of the femoral head was another severe complication. The high incidence of osteonecrosis of the femoral head after early capsular arthroplasty is generally believed to be caused by the high intra-articular pressure after traction reduction at the first stage. As reported by Chung *et al.*, a total of 41 patients who underwent capsular arthroplasty (73.2%) had some evidence of osteonecrosis [12]. Later, Bertrand and Stans stopped performing one-stage traction and replaced it with femoral shortening osteotomy, resulting in a significant decrease in the incidence of femoral head osteonecrosis [24, 25]. The lack of correct understanding of the blood supply of the femoral head is also one of the causes of femoral head necrosis. With the application of accurate perceptions of femoral head perfusion [26], all nine patients reported by Ganz had no femoral head necrosis. In our study, four cases of femoral head necrosis occurred (4.3%), which mostly occurred in the early period and may be caused by inexperience with surgical techniques or extra operations.

Due to the small sample size reported previously, the spectrum of indications for surgery has not been definitely established. In the present study, we found that capsular thickness had a great impact on both post-operative functional and radiographic outcomes. In addition, the capsular quality also affected the radiographic results. The earlier reports did not provide a detailed analysis of the importance of hip capsular quality. In addition, our results also showed that the new socket diameter and femoral head size were predictive factors for hip function. The larger the diameter of the femoral head is, the lower the risk of hip dysfunction. Therefore, patients with a femoral head diameter smaller than that of the normal side are not suitable for capsular arthroplasty. Because of the complexity of surgical techniques, the clinical results of capsular arthroplasty can be affected by the learning curve of this procedure. The surgeries in our series of cases were conducted over a span of approximately 10 years. Complications and poor hip function mostly occurred early in the study period, which was caused by inexperience with either the surgical technique or the selection of indications. Little is known about the importance of capsular quality and femoral head size. However, the present study failed to identify the cutoff values for femoral head size or capsular thickness due to the relatively small sample size. Although our statistical analysis showed that older age was not a predictive factor of failure, in our clinical practice, we found that poor results were more common in patients older than 20 years old. Therefore, our indications for the modified capsular arthroplasty are as follows: 8–20 years old, unilateral hip dislocation (Hartofilakidis Types II and III), magnetic resonance imaging (MRI) showing good cartilage of the femoral head, continuous and uniform signal of the capsule, normal size of the femoral head and compliance with regular rehabilitation.

Our study has some limitations. First, 22/92 hips were followed up for less than 2 years. Longer-term follow-up is needed due to the long rehabilitation time post-operatively. The second limitation is that some patients did not return to follow-up at the required time, and the discrepancy in patient compliance with rehabilitation exercise may result in joint stiffness or osteoarthritis. Therefore, it is difficult to analyze the influence of various rehabilitation exercises on clinical results. Third, bias may arise, as the hip function of patients who were unable to return for review every time was evaluated by mobile phone or Internet. The remote patient revisit could assess gait, functional questionnaires and radiographs, but not the range of motion, muscle strength or other specialized physical examinations. Fourth, the gait analysis system was not available in our study, so the gait was evaluated qualitatively. Finally, the acetabular sourcil was indistinct in most X-rays at the final follow-up; thus, it was difficult to evaluate radiographs quantitatively by the lateral center-edge angle or acetabular index angle.

## CONCLUSIONS

We confirmed the efficacy of the modified capsular arthroplasty in the treatment of young patients with developmental hip dislocation. We emphasize the importance of capsular quality and recommend that pre-operative MRI be routinely used to determine capsule quality and thickness. With a suitable selection of indications, iHOT-12 can be significantly improved, and more than 80% of patients can obtain excellent mHHS. This procedure can restore the rotation center of the hip joint with a low incidence of femoral head necrosis or severe osteoarthritis. There is a certain incidence of complications, mainly joint stiffness. Regular rehabilitation exercises are of great importance after surgery.

## Data Availability

The data underlying this article will be shared on reasonable request to the corresponding author.
